# Application of Pulsed Electric Field in Antifouling Treatment of Sodium Gluconate Mother Liquor by Electrodialysis

**DOI:** 10.3390/ma13112501

**Published:** 2020-05-30

**Authors:** Qi Gao, Zichao Li, Chunxiao Lei, Rongqiang Fu, Wei Wang, Qun Li, Zhaoming Liu

**Affiliations:** 1 College of Chemistry and Chemical Engineering, Qingdao University, Qingdao 266071, China; 17864228621@163.com (Q.G.); Thunder_cx@163.com (C.L.); 2 College of Life Sciences, Qingdao University, Qingdao 266071, China; zichaoli@qdu.edu.cn; 3 Key Laboratory of Charged Polymeric Membrane Materials of Shandong Province, Shandong Tianwei Membrane Technology Co., Ltd., Weifang 261061, China; rongqiangfu@gmail.com (R.F.); ww@sdtianwei.com (W.W.); 4 State Key Laboratory of Bio-Fibers and Eco-Textiles, Shandong Collaborative Innovation Center of Marine Biobased Fibers and Ecological Textiles, Qingdao University, Qingdao 266071, China

**Keywords:** pulsed electric field, electrodialysis, sodium gluconate, membrane fouling

## Abstract

Contamination of ion exchange membranes is one of the major problems in electrodialysis. Among the solutions that have been proposed and tested to alleviate membrane fouling during electrodialysis so far, applying a pulsed electric field (PEF) at a fixed application time (T_on_) followed by a pause time (T_off_) has been proved to be effective. In this study, the PEF was applied to desalinate sodium gluconate mother liquor by ED. The experimental properties of conventional ED and pulsed ED and their effects on membrane fouling were compared. The results show that compared with conventional ED, pulsed ED can alleviate concentration polarization and enhance the performance of ED. Similarly, in the process of continuous batch treatment of mother liquor under the PEF condition, large organic molecules can be effectively prevented from depositing on the membrane surface. Therefore, an anion exchange membrane (AEM) under the condition of PEF is contaminated mainly by organic molecules with a relatively smaller size. Both the surface and interior of AEM membrane were affected by organic pollutants under conventional electric field (CEF) conditions.

## 1. Introduction

Sodium gluconate is produced by fungal fermentation, and the mother liquid contains sodium gluconate and various organic species due to addition of nutrients for cell growth and occurrence of cell metabolites [1]. Sodium gluconate can be used as a highly effective chelating agent in construction, as a surface cleaner for steel and iron, a bottle cleaner, aluminum oxide coloring for electroplating industry, and as a highly effective retarder and water reducer for concrete industry [2]. Mother liquor can be used as animal feed after desalination to avoid environmental pollution. From the economic and environmental point of view, it is necessary to find a suitable method to recover sodium gluconate from mother liquor. Electrodialysis (ED) is a method to separate salts from other components under the action of electric field without chemical consumption or waste generation. It has played an important role in various sewage purification, sea water desalination and salt production [3,4,5]. It could be used to demineralize mother liquid to recover sodium gluconate.

Contamination of ion exchange membrane is a serious problem in ED treatment [6]. Fouling is mainly attributed to organic matter, minerals, colloids, biomass and particles absorbed in the membrane and deposited on the membrane surface [7,8,9]. Usually, due to the chemical or physical interaction between the membrane and the pollutants, the membrane resistance increases, and the membrane selectivity reduces, resulting in a higher energy consumption and a lower efficiency than those of the original state [4,6,10,11].

Many methods have been investigated to alleviate membrane fouling in ED, including pretreatment of the feed solution, optimization of process conditions and modification of the membrane properties. Although the above methods can partially alleviate membrane fouling and improve the performance of ED, it is still necessary to clean the equipment in the practical application process, such as chemical cleaning [12]. It has been found that pulsed ED can reduce concentration polarization, improve membrane selectivity and alleviate membrane fouling, thus significantly improving the performance of ion exchange membrane process [13]. The process of pulsed ED consists of application of consecutive pulse and pause lapses of a certain duration (T_on_/T_off_).

In this study, pulsed electric fields (PEF) was used in ED experiment as a method to alleviate fouling. The application of PEF in ED, especially when the suspension period is prolonged, results in electrophoretic motion on the membrane surface [14]. During the suspension, the solution concentration at the interface of the ion exchange membrane will be partially restored, so the concentration polarization will be relieved. In addition, it has been reported that the application of PEF in the ED process can enhance ion migration owing to the alleviated concentration polarization [15,16,17]. In 1995, Karlin and Kropotov first proposed the use of PEF to control ion migration during ED to separate Na^+^ and Ca^2+^. The pulse mode consists of a fixed time for applying a constant current and a pause time for energizing, which is the applied hash current. The method of Karlin and Kropotov was based on reducing concentration polarization on the membrane surface [18]. It has been proved that the desalination can be strengthened several times according to the pulse-pause characteristics of PEF [15,19]. The application of PEF in the ED process can also effectively alleviate the fouling problem of fermentation wastewater, which mainly reduces the fouling degree of organic matter to the ion exchange membrane [7,12]. The beneficial impact of using PEF on scaling remission during ED experiment of model salt solutions has been successfully demonstrated [20,21,22]. The purpose of this study is to use PEF to alleviate the fouling of sodium gluconate mother liquor during desalination and to reduce the fouling rate of ion exchange membrane. The effects of PEF on the performance of ED and membrane fouling were studied by comparing conventional ED with pulsed ED.

## 2. Experimental

### 2.1. Materials

Sodium gluconate mother liquor was kindly supplied by Shandong Fuyang Biotechnology Co., Ltd. (Fuyang, China). Microfiltration pretreatment was applied to remove suspended solids in the mother liquor before experiment. Both the anion exchange membranes (AEM, product code TWEDA1) and the cation exchange membranes (CEM, product code TWEDC1) were from Shandong Tianwei Membrane Technology Co., Ltd. (Weifang, China). The characteristics of these membranes are shown in Table 1. The chemical reagent NaCl (AR grade) used was from Sinopharm Group Chemical reagent Co., Ltd. (Jinan, China). The water used in the experiment was deionized.

### 2.2. Setup

**Figure 1:**
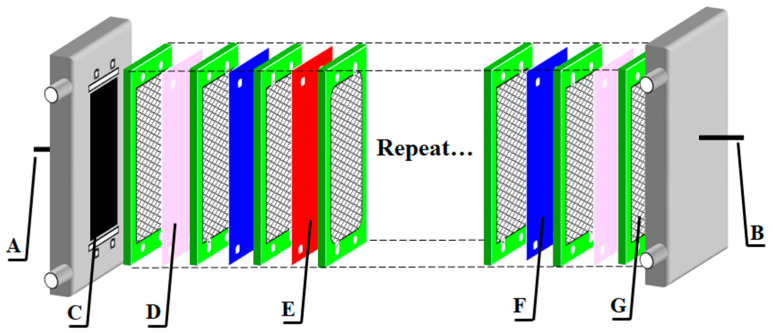
Internal structure of the electrodialysis (ED) membrane stack. (A) cathode; (B) anode; (C) electrode; (D) special cation exchange membranes (CEM); (E) CEM; (F) anion exchange membranes (AEM); (G) compartment.

The laboratory membrane stacks, supplied by Shandong Tianwei Membrane Technology Co., Ltd., were applied in the experiment. The internal structure of the ED stack is shown in Figure 1. The repeating unit consisted of two ion exchange membranes (AEM and CEM) and two spacers. The 9 pieces of CEMs and 10 pieces of AEMs made up 10 repeating units of the membrane stack. In addition, 2 pieces of special CEMs were set near the electrode compartments. Titanium plates coated with Ta/Ir catalyst were used as the electrodes. The effective area of each membrane was 84 cm^2^, and the thickness of each compartment was 0.5 mm [23].

**Figure 2:**
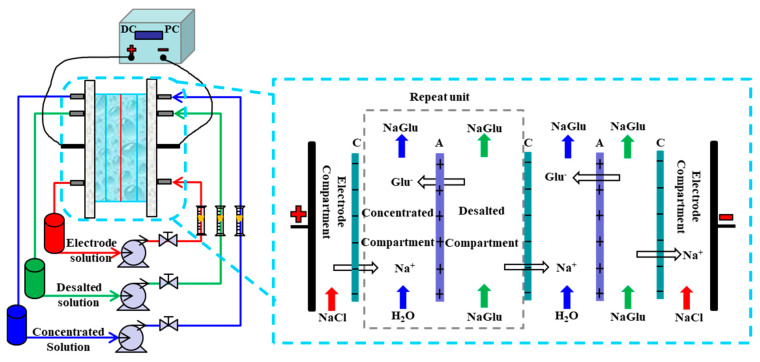
Schematic representation of the ED applied to separation sodium gluconate from the sodium gluconate mother liquor. A: AEM, C: CEM.

As shown in Figure 2, the ion exchange membranes divide the membrane stack into three independent compartments (desalted compartment, concentrated compartment and electrode compartment). Each compartment was connected to a separated external liquid container, and the liquid circulated in the container and compartment. The magnetic drive pumps (MG200/DC24W1, Nanjing Ouerike Micro Pump Co., Ltd., Nanjing, China) were used to recirculate the solutions. The direct-current power supply (GPS-4303C/4302C, Good Will Instrument Co., Ltd. and the pulse current power supply (HSP3010, Shenzhen Henghui Electronics Co., Ltd., Shenzhen, China) were connected to the electrodes to provide electric current.

### 2.3. Experimental Operational Conditions

The dilute compartment was fed with 1 L pretreated mother liquor, the concentrated compartment was fed with 0.5 L deionized water and 1% NaCl solution was used as the electrode rinse. The linear flow rates of the concentrate and dilute were 3 cm/s, and those of electrode solutions were 6 cm/s.

**Figure 3:**
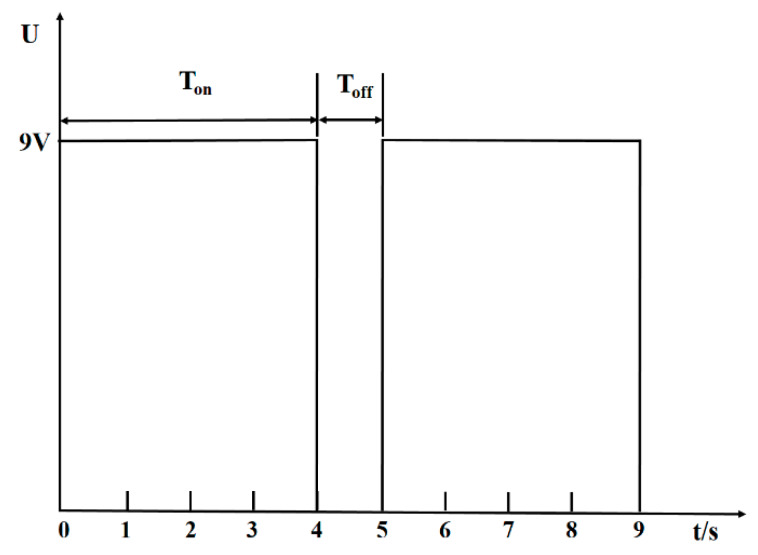
Schematic diagram of the pulsed electric field (PEF) pattern. U: constant voltage, T_on_ = 4 s, T_off_ = 1 s.

The conventional ED and the pulsed ED were used to desalinate sodium gluconate mother liquor. Two membrane stacks were adopted to the experiment, which have the exact same inner structure and ion exchange membrane. Each group of desalination experiments was run in 5 batches at a constant voltage of 9 V. The effective time of each group of experiments was 160 min, so the conventional ED experiment was stopped at 160 min. As shown in Figure 3, the pulsed mode consisted of a constant voltage during a fixed time (T_on_ = 4 s) was followed by a pause duration (T_off_ = 1 s). The effective time under the PEF condition was after deducting the pause time, so the effective time of the PEF experiment was 160 min with the total running time 200 min. The pH value of the mother solution of sodium gluconate was 5.47, and the pH value remained basically unchanged between 5.4 and 5.5 in the experiment under different electric field conditions.

### 2.4. Analytical Methods

#### 2.4.1. Measurement of Stack Resistance

According to Ohm’s Law, the resistance value of the stack was calculated directly by the voltage and current from the power supply [24].

#### 2.4.2. Production Energy Consumption and Salt Flux

Production energy consumption was the energy consumption per unit volume of sodium gluconate mother liquor treated by ED according to the following equation:

(1) $$E=\frac{\int_{0}^{t}UIdt}{V}$$

where *U* is the voltage (V); *I* is the current (A); *t* is the time of the ED experiment (h); *V* is the volume of the final diluate (t).

The salt flux is defined as:

(2) $$C=\frac{V\cdot c}{A\cdot t}$$

where *V* is the volume of the concentrate; *c* is concentration of the concentrate; *A* is the effective area of the membrane; *t* is the effective time of the ED experiment.

#### 2.4.3. Current Density

The current density (*J*) represents its value at a certain time under constant pressure and the average current density (*J*_0_) represents its average value over a period under constant pressure, that is, the current flow through the membrane per unit area expressed as A/m^2^.

Equation for calculating current density:

(3) J=I/A

where *I* is the current entering the ED device (A); *A* is the effective area of the membrane (m^2^).

Equation for calculating average current density:

(4) $$J_{0}=\frac{\int_{0}^{t}Idt}{A\cdot t}$$

where *I* is the current entering the ED device (A); *t* is the duration of the experiment (s); *A* is the effective area of the membrane (m^2^).

#### 2.4.4. Conductivity Control

Conductivity was measured using conductivity meter (DDS-11A conductometer, Shanghai, China). Conductivity can be used to represent salt concentration [25]. Thus, the demineralization rate is defined as:

(5) $$DR=\frac{\sigma_{i}-\sigma_{f}}{\sigma_{i}}\times 100\%$$

where *DR* is the demineralization rate (%); $\sigma_{i}$ is the initial conductivity of the diluate; $\sigma_{f}$ is the final conductivity of the diluate (mS/cm).

### 2.5. Fouling Analysis

#### 2.5.1. Membrane Electric Resistance

The membrane electric resistance was tested with a four-chamber testing cell [6]. The effective area (S) of the membrane being tested was 7 cm^2^. A set of Ag/AgCl reference electrode was installed, closed to the membrane for measuring the voltage drop across the membrane. The electrolyte was 0.5 M NaCl and the direct-current (I) of 50 mA was used during the test. The test system was placed in a 25 °C thermostat. The membrane electric resistance is defined as:

(6) $$R=\frac{\left(E_{1}-E_{2}\right)}{I}\cdot S$$

where *R* is the membrane electric resistance (Ω·cm^2^); $E_{1}$ is the reference electrode potential difference with the membrane in the device (mV); $E_{2}$ is the reference electrode potential difference without the membrane (mV); *I* is the electric current (mA); *S* is the effective area of the membrane (cm^2^).

#### 2.5.2. Scanning Electron Microscope

Images were taken on new and fouled membrane pieces to examine the fouling degree of membranes at 9 kV with a scanning electron microscopy (SEM; TM-3030, Hitachi High-Technologies Corporation, Tokyo, Japan).

## 3. Results and Discussion

### 3.1. Effect of Two Electric Fields on ED Treatment of Mother Liquor

**Figure 4:**
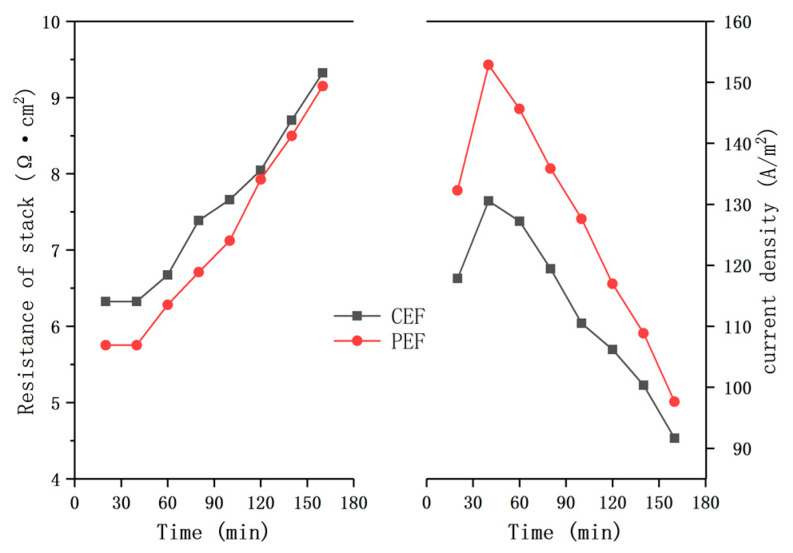
Membrane stack resistance and current density in the demineralization of sodium gluconate mother liquor. The conventional direct-current power and the pulse direct-current power operated at constant voltage of 9 V.

The demineralization of sodium gluconate mother liquor was performed at a constant voltage of 9 V. The ED performances between conventional electric field (CEF) and PEF were compared in terms of the stack resistance, current density, solution conductivity and demineralization rate. The stack resistance and current density of different electric field conditions with time are shown in Figure 4. The membrane stack of CEF showed somewhat higher resistance and lower current density than that of PEF. There exists the concentration polarization in ED process. The solution concentration near membranes surface in the dilute compartment gets lower. There is no voltage output in the pulse ED during the pause stage, which can partially restore the concentration on the surface of the ion exchange membrane and alleviate the concentration polarization phenomenon. Therefore, compared with conventional ED, pulsed ED has a higher ion concentration on the surface of the ion exchange membrane, which reduces the resistance of experimental system and results in a higher current density [26].

**Figure 5:**
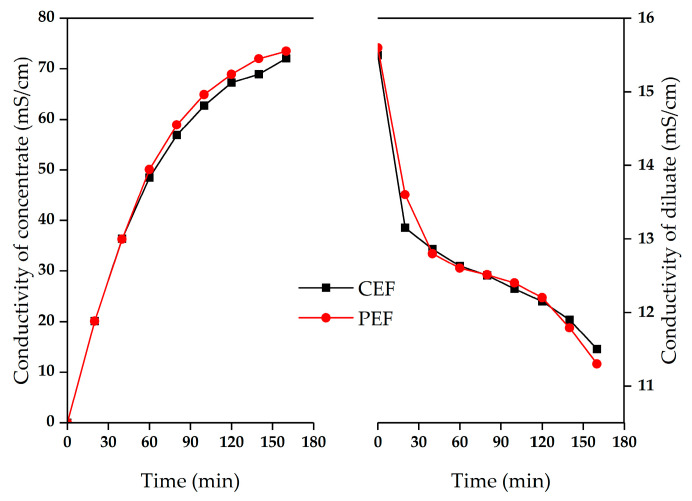
Conductivity of the concentrate and the dilute in the demineralization of sodium gluconate mother liquor. The conventional direct-current power and the pulse direct-current power operated at constant voltage of 9 V.

Figure 5 illustrates the conductivity of concentrate and diluate for the different electric field conditions as the time increased. At the end of the experiments, pulse ED has the higher conductivity of the concentrate and the lower conductivity of the dilute, indicating the mother liquor treated by pulse ED had better desalting effect. As shown in Figure 4, the resistances of two membrane stacks were relatively stable for the first 40 min, then the resistances started to increase. In addition, the current density of the two membrane stacks first increased with time, and then decreased linearly. Similarly, the conductivity of the concentrate in Figure 5 increased linearly for the first 40 min, but afterward the slopes decreased. The conductivity of diluate presents an opposite trend. Bazinet et al. and Perez et al. ’s works have shown that, according to Equation (4), the desalination rate can be calculated by the initial and final conductivity values of the diluate [27,28]. The demineralization rates were 25.8% in conventional ED and 27.56% in pulsed ED, respectively. The experimental results showed that no membrane fouling was observed in the desalination experiment of mother liquor treated by ED in batch 1. The ED performance of sodium gluconate mother liquor treated by pulsed ED was slightly better than that of CEF. The fouling of the ion exchange membrane was attributed to the deposition of organic matter on the membrane surface due to the adsorption of the membrane surface. In addition, the transport of organics having low electric mobility through membranes gave more fouling effect inside of membrane and on the membrane surface [24].

### 3.2. Fouling Phenomena in ED of Sodium Gluconate Mother Liquor

In order to study the fouling phenomenon of membrane stack, five batches of sodium gluconate mother liquor were repeatedly treated by conventional ED. The experimental conditions of ED were the same in the 5 batches. Under CEF conditions, the experimental properties of 5 batches of ED were compared, as shown in Table 2. The resistance and production energy consumption of the membrane stack were 9.3 Ω·cm^2^ and 185.0 kWh/t in batch 1, respectively, while they were 25.3 Ω·cm^2^ and 252.4 kWh/t in batch 5. It can be seen from Table 2 that the resistance value and production energy consumption of the membrane stack increased with the batch. The decrease of current efficiency, current density and demineralization rate in Table 2 indicates that the performance of ED is decreasing. The increased resistance and energy consumption should be caused by the membrane fouling. It is generally believed that the reason for the increase of resistance in the ED process is the generation of concentration polarization caused by ion depletion and the membrane fouling caused by fouling deposition on the membrane surface [25]. Therefore, in this study, after 5 batches of repeated ED mother liquor experiments, foulants in the mother liquor fouled the ion exchange membranes, resulting in increased resistance and production energy consumption of the membrane stack.

In order to study the fouling degree of the membrane, the membrane stack was disassembled after the ED experiments to test the resistance value of the ion-exchange membranes. Then the tested membranes were chemically cleaned. AEMs and CEMs were first soaked in 1 L hydrochloric acid (1%) for 12 h, rinsed thoroughly with deionized water, soaked in 1 L sodium hydroxide (1%) for 12 h and finally rinsed thoroughly with deionized water. The membrane resistance value was the average of three repeated test data. Generally, the fouling of the membrane surface by fouling deposition is called reversible fouling that can be removed by chemical cleaning. At the same time, irreversible foulant was difficult to remove with cleaning fluid [29]. As shown in Table 3, the resistance of the contaminated AEMs after conventional ED treatment increased by 81.2% compared with the new membranes, and the resistance of the cleaned membranes increased by 27.35%. As can be seen from the table, the dirt on the membranes can be removed with most of the cleaning foulant. The foulant of mother liquor in ED is mainly caused by reversible foulant. In addition, it was noted that the resistance of CEMs decreased slightly compared to the new membranes. It is inferred that the internal structure of the ion exchange membranes may be more porous than that of the new membranes after prolonged immersion, resulting in lower resistance and selectivity. Wang et al. observed similar results in their experiments [6]. In Table 2, there was a slight decrease in resistance and production energy consumption for the first three batches. In combination with the change analysis of the membrane resistance in Table 3, it may be that the structure of the ion exchange membrane became loose after soaking for a long time, resulting in a slight change in the overall resistance of the membrane stack. However, the first three batches will also be affected by membrane or membrane surface contamination, and the reduction of resistance caused by membrane structure looseness will partially offset the increase in resistance caused by membrane fouling. From the fourth batch, the porosity of the membrane structure reached the limit and membrane fouling continued to occur, thus the membrane resistance between the third batch and the fourth and fifth batches rose sharply. Based on electrostatic interaction, negatively charged organic substances tend to be adsorbed on the surface of AEMs and repelled by negatively charged CEMs [11]. Therefore, organic substances in mother liquor were easy to foul AEMs, and CEMs was not fouled basically.

### 3.3. Pulse ED Treatment of Sodium Gluconate Mother Liquor to Reduce Fouling

**Figure 6:**
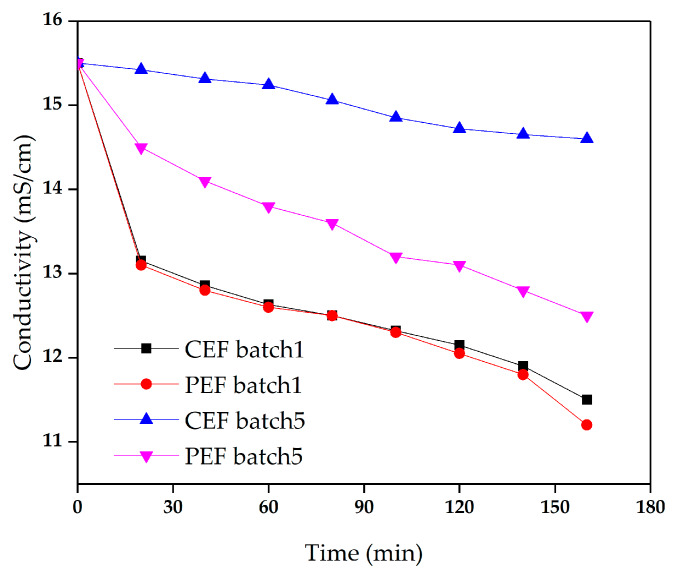
The conductivity of the dilute in the demineralization of sodium gluconate mother liquor in batch 1 and batch 5. The conventional direct-current power and the pulse direct-current power operated at constant voltage of 9 V.

**Figure 7:**
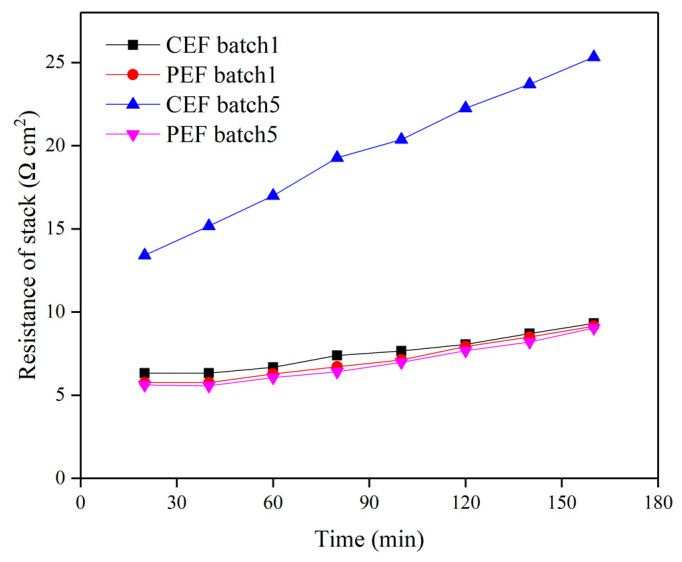
Membrane stack resistance in the demineralization of sodium gluconate mother liquor in batch 1 and batch 5. The conventional direct-current power and the pulse direct-current power operated at constant voltage of 9 V.

Figure 6 shows the conductivity of the dilute in batch 1 and batch 5 for conventional ED and pulse ED. In batch 1, the conductivity curves were almost overlapped. However, in batch 5, the conductivity of CEF showed a slightly lower decrease rate than the PEF. In addition, the PEF influence on the membrane performance can be observed in the graph of membrane stack resistance with time, as shown in Figure 7. The resistance of CEF in batch 5 was significantly higher than those in other conditions. After the CEF reactor was run for 5 batches, the ED performance decreased obviously, and the organic pollutants adsorbed on the AEM slowed down the transmission of anions through the membrane [25].

**Figure 8:**
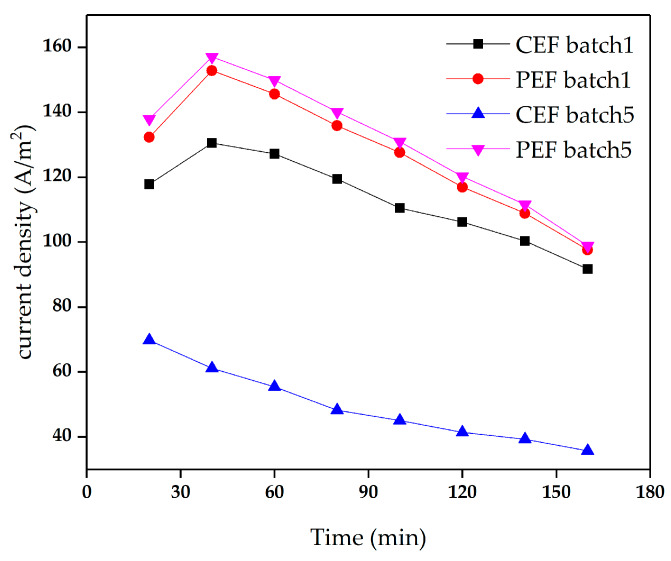
The current density in the demineralization of sodium gluconate mother liquor in batch 1 and batch 5. The conventional direct-current power and the pulse direct-current power operated at constant voltage of 9 V.

As shown in Figure 8, there was almost no significant difference between the current density of batch 1 and batch 5 for pulse ED. In conventional ED, however, major differences were observed between the initial batch and the final batch, with a significant overall decrease in current density after 5 runs. After 5 times of treatment with conventional ED, the membrane fouling became more and more serious, increasing the membrane resistance and reducing the mobility of ions through the membrane, resulting in a significant decrease in current density. The ED performances of PEF are listed in Table 4. It can be clearly observed that the demineralization rate and production energy consumption under PEF are 19.35% and 193.47 kWh/t, respectively, compared with 5.8% and 252.37 kWh/t under CEF, respectively. Therefore, after running sodium gluconate mother liquor for many times, the ED performance under PEF was better than that of CEF.

**Figure 9:**
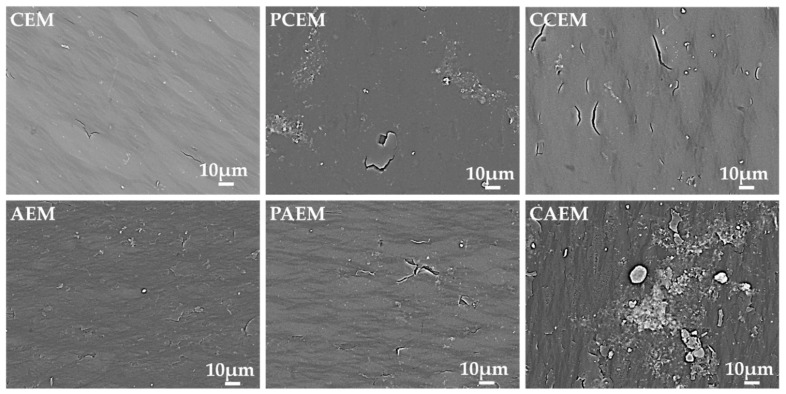
Scanning electron microscopy (SEM) images of cation membranes and anionic membranes after five batch ED and pulse ED runs: CEM and AEM, unused ion- exchange membranes; Cation exchange membranes under pulsed electric field conditions (PCEM) and Anion exchange membranes under pulsed electric field conditions (PAEM), ion exchange membranes used for pulse ED; Cation exchange membranes under conventional electric field conditions (CCEM) and Anion exchange membranes under conventional electric field conditions (CAEM), ion-exchange membranes used for ED.

As shown in Table 3, the AEM resistance treated under PEF increased by 22.9% compared with the new membrane resistance, while the AEM resistance after cleaning increased by 18.4%. However, the resistance of the CEM is basically unchanged. The resistance value of the contaminated AEM increased by 27.35% compared with the new membrane under the CEF condition. To assess the contamination of the membrane surface, scanning electron microscopy (SEM) images were taken of the anionic membrane surface and the cationic membrane surface. Figure 9 shows the surface morphology of the ion exchange membrane. Compared with the clean and intact surface of the original cationic membrane, the AEMs under CEF conditions (CAEM) were found to be agglomerated and flocculent. Studies have shown that organic pollutants contaminate AEM based on electrostatic interactions and geometric interactions between pollutants and membranes [30]. The negatively charged organic matter tends to foul the positively charged AEMs, resulting in increased membrane electric resistance. The relatively smaller organic molecules were trapped in the matrix of the membrane compared with the ion channels in the membrane. On the contrast, the larger organic molecules can be hindered into the membrane, resulting in deposition on the membrane surface [29]. The results showed that both the surface and interior of AEM membrane were affected by organic pollutants under CEF condition. In the process of pulse suspension, the feed liquid is disturbed and has a certain washing effect on the membrane surface [26]. According to the types of pollutants, the types of membrane fouling include colloidal fouling, organic fouling, inorganic fouling and biologic fouling [30]. Before the electrodialysis experiment, sodium gluconate mother liquor was pretreated and the suspension, bacteria and colloid in mother liquor were removed by microfiltration membrane. Thereby, membrane fouling is not colloid fouling or biologic fouling [31]. After the electrodialysis experiment, the contaminated membrane was cleaned with hydrochloric acid. Since the membrane resistance hardly changes before and after cleaning, it can be inferred that there was no inorganic contamination in the membrane fouling. However, small molecular weight organics trapped in the membrane are not easily washed down, resulting in a small amount of irreversible contamination. Therefore, the AEM is mainly contaminated by the organic molecules with the relatively smaller size.

## 4. Conclusions

In this study, demineralization of sodium gluconate mother liquor was performed by ED. It was observed that the pulse ED could reduce fouling potentials and improve ED performances compared with the conventional ED. It is considered that the pulse ED in the treatment of mother liquor disturbs the diffusion layer on the membrane surface, alleviating the concentration polarization phenomenon and decreasing the electric resistance of the membrane stack. The results showed that both the surface and interior of AEM membrane were affected by organic pollutants under CEF condition. The organic matter with the relatively larger size in the mother liquor deposited on the surface of the AEM mainly through adsorption and the smaller organic matter can be trapped in the matrix of the membrane. In addition, the PEF was effective in preventing the deposition of the larger organic matter on the membrane surface. However, for organic matter with small molecular size, it is easy to be trapped in the membrane, leading to irreversible fouling. The AEM under the condition of PEF is mainly contaminated by the organic molecules with the relatively smaller size.

## Figures and Tables

**Table 1:** The main characteristics of anion exchange membranes (AEM) and cation exchange membranes (CEM) used in the experiments.

Membrane Type	Thickness/Wet (µm)	IEC ^a^ (Ion Exchange Capacity, meq·g^−1^)	Area Resistance ^b^ (Ω·cm^2^)	Water Uptake ^c^ (%)	Transport Number ^d^
AEM	40–50	0.90–1.10	≤2.5	15–20	≥0.98
CEM	40–50	0.90–1.10	≤3.3	15–20	≥0.97

The data were collected from the product brochure provided by manufacturers. ^a^ Ion exchange capacity test conditions: relative to the dry membranes, 25 °C; ^b^ Area resistance of Area resistance of AEM and CEM was measured as Cl^−^ and Na^+^ form in 0.5 M NaCl at 25 °C, respectively; ^c^ Water uptake was determined by weight ratio of absorbed water to membrane dry weight; ^d^ Transport number of AEM and CEM was measured as Cl^−^ and Na^+^ form in 0.5 M and 0.1 M NaCl solution at 25 °C, respectively.

**Table 2:** Conventional ED performance of sodium gluconate mother liquor.

	Batch 1	Batch 2	Batch 3	Batch 4	Batch 5
Current efficiency	46.5%	48.2%	51.7%	39.7%	37.0%
Average current density (A/m^2^)	113.3	114.6	116.1	69.5	49.6
Demineralization rate	25.80%	24.58%	25.67%	12.90%	5.80%
Production energy consumption (kWh/t)	185.1	178.7	165.4	226.9	252.4
Resistance of the membrane stack (Ω·cm^2^)	9.3	9.2	9.1	21.0	25.3

**Table 3:** Electric resistance of AEMs and CEMs before and after fouling under different electric field conditions.

Electric Resistance (Ω cm^2^)		AEM	CEM
Conventional electric field (CEF)	R_0_^1^	2.34	3.24
	R_1_^2^	4.24	3.17
	ΔR_1_^4^	81.20%	−2.16%
	R_2_^3^	2.98	3.15
	ΔR_2_^5^	27.35%	−2.78%
Pulsed electric field (PEF)	R_0_	2.45	3.21
	R_1_	3.01	3.13
	ΔR_1_	22.86%	−2.94%
	R_2_	2.96	3.09
	ΔR_2_	20.81%	−3.74%

^1^ R_0_: the electric resistance before fouling; ^2^ R_1_: the electric resistance after fouling; ^3^ R_2_: the electric resistance after cleaning; ^4^ ΔR_1_ = (R_1_ − R_0_)/R_0_; ^5^ ΔR_2_ = (R_2_ − R_0_)/R_0_.

**Table 4:** Pulse ED performance of sodium gluconate mother liquor.

	Batch 1	Batch 2	Batch 3	Batch 4	Batch 5
Current efficiency	42.6%	46.4%	46.3%	47.5%	46.8%
Average current density (A/m^2^)	127.5	128.2	129.7	130.4	131.3
Demineralization rate	28.60%	27.09%	26.4%	24.51%	19.35%
Production energy consumption (kWh/t)	199.1	194.0	195.6	190.0	193.5
Resistance of the membrane stack (Ω·cm^2^)	9.2	9.2	9.1	9.0	9.0

## Abbreviations

ED	Electrodialysis
PEF	Pulsed electric field
CEF	Conventional electric field
AEM	Anion exchange membrane
CEM	Cation exchange membrane
CAEM	Anion exchange membranes under conventional electric field conditions
PAEM	Anion exchange membranes under pulsed electric field conditions
CCEM	Cation exchange membranes under conventional electric field conditions
PCEM	Cation exchange membranes under pulsed electric field conditions
SEM	Scanning electron microscopy
IEC	Ion exchange capacity
